# CoReHA 2.0: A Software Package for *In Vivo* MREIT Experiments

**DOI:** 10.1155/2013/941745

**Published:** 2013-02-24

**Authors:** Kiwan Jeon, Chang-Ock Lee

**Affiliations:** ^1^National Institute for Mathematical Sciences, Daejeon 305-811, Republic of Korea; ^2^Department of Mathematical Sciences, KAIST, Daejeon 305-701, Republic of Korea

## Abstract

Magnetic resonance electrical impedance tomography (MREIT) is a new medical imaging modality visualizing static conductivity images of electrically conducting subjects. Recently, MREIT has rapidly progressed in its theory, algorithm, and experiment technique and now reached to the stage of *in vivo* animal experiments. In this paper, we present a software, named CoReHA 2.0 standing for the second version of conductivity reconstructor using harmonic algorithms, to facilitate *in vivo* MREIT reconstruction of conductivity image. This software offers various computational tools including preprocessing of MREIT data, identification of 2D geometry of the imaging domain and electrode positions, and reconstruction of cross-sectional scaled conductivity images from MREIT data. In particular, in the new version, we added several tools including ramp-preserving denoising, harmonic inpainting, and local harmonic *B*_*z*_ algorithm to deal with data from *in vivo* experiments. The presented software will be useful to researchers in the field of MREIT for simulation, validation, and further technical development.

## 1. Introduction

Recently, a new imaging modality called magnetic resonance electrical impedance tomography (MREIT) has been introduced, which allows high resolution imaging of tomographic electrical conductivity distributions of biological objects [1, 2]. The technique involves (i) current injection into an electrically conducting object such as animal or human body through surface electrodes, (ii) measurement of induced internal magnetic flux density using an MRI system, typically only the *z*-component *B*_*z*_ of the induced magnetic flux density **B** = (*B*_*x*_, *B*_*y*_, *B*_*z*_), where *z* is the axis parallel to the main magnetic field of the MR scanner, and (iii) conductivity reconstruction by solving nonlinear boundary value problems with given injected currents and measured magnetic flux density, employing finite element methods. Although it looks straight forward to reconstruct conductivity distribution as described in the three steps, computation involves several innovative approaches including magnetic flux density estimation, data verification, segmentation, and solving forward/inverse problems. These specific computations cannot be handled through readily available finite element packages; thus, it is required to develop a user friendly software with graphics user interface (GUI) for those who wish to reconstruct conductivity distributions. Upon these requests, we developed a software package, called CoReHA [3, 4] which stands for conductivity reconstructor using harmonic algorithms, using VC++ MFC 6.0 (Microsoft Foundation Class Library 6.0) and OpenGL under the Microsoft Windows operating system. Based on the harmonic *B*_*z*_ algorithm [5], CoReHA supports all procedures from the preprocessing of raw data to conductivity imaging through the intuitively apprehensible graphic user interface, more specifically, data conversion of raw *k*-space data, data verification, segmentation tools for numerical computation, solvers of forward/inverse problems using finite element methods, and 2D/3D data view as well as histogram. This software has been a major tool to facilitate multilateral studies for MREIT and has brought out successful reconstruction of conductivity imaging in many researches [4, 6–8].

In the stage of *in vivo* MREIT experiments, we have to address the improvement of the signal-to-noise ratio (SNR) of measured *B*_*z*_ data, since *B*_*z*_ data has weak strength due to the low amount of injected current for the safety guide. Moreover, there may exist MR signal void regions in the animal or human body, where noise levels of *B*_*z*_ are excessively high, and as a result, uncertain effects are produced. For dealing with these technical problems, several methods were developed, called ramp preserving denoising [9], harmonic inpainting [10], and local harmonic *B*_*z*_ algorithm [11]. Incorporating these new features, we release the second version of CoReHA for *in vivo* MREIT experiments. The presented software will be useful to researchers in the field of MREIT experimental studies as well as *in vivo* animal/human experiments. CoReHA is available from the website http://iirc.khu.ac.kr/.

This paper is organized as follows. In Section 2, MREIT system is introduced and related works are explained. Brief explanation of the previous version of this software is given for comprehensive understanding of our works in Section 3. Details of new tools of the software for dealing with *in vivo* stage issues are provided in Section 4. Finally, we close in Section 5 with conclusions and a discussion of future works.

## 2. The Basic of MREIT and Related Works

**Figure 1:**
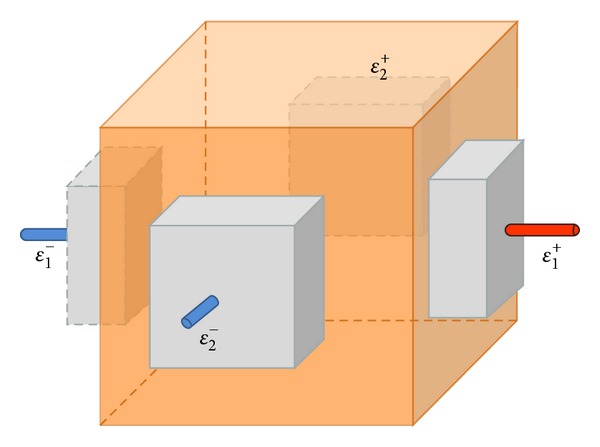
Domain *Ω* for MREIT.

We briefly explain how MR scanner is used as a tool to capture internal magnetic flux density images, and the MR scanner has its magnetic field in *z*-direction. Let an electrically conducting subject occupy a three-dimensional domain *Ω* with its boundary ∂*Ω* and a conductivity distribution *σ*. As shown in Figure 1, we attach pairs of electrodes *ℰ*_*j*_^+^ and *ℰ*_*j*_^−^ along ∂*Ω* in order to inject a current *I*_*j*_ in a form of pulses whose timing is synchronized with an MR pulse sequence for *j* = 1,2. The injection current produces current density **J**_*j*_ satisfying the following elliptic equations:

(1) $$\begin{array}{c} \nabla \cdot \left(\sigma \nabla u_{j}\left[\sigma \right]\right)=0\;\text{in}\;\;\Omega ,\\ I=\int_{{ℰ}_{j}^{+}}\sigma \frac{\partial u_{j}\left[\sigma \right]}{\partial n}ds=-\int_{{ℰ}_{j}^{-}}\sigma \frac{\partial u_{j}\left[\sigma \right]}{\partial n}ds,\\ \nabla u_{j}\left[\sigma \right]\times n{|}_{{ℰ}_{j}^{+}\cup {ℰ}_{j}^{-}}=0,\\ \sigma \frac{\partial u_{j}\left[\sigma \right]}{\partial n}=0\;\mathrm{on}\;\;\partial \Omega \setminus \bar{{ℰ}_{j}^{+}\cup {ℰ}_{j}^{-}}, \end{array}$$

where **J**_*j*_ = −*σ*∇*u*_*j*_. Also, magnetic flux density **B** is given by Biot-Savart law as follows:

(2) $$\begin{array}{c} B\left(x\right)=\frac{\mu_{0}}{4\pi }\int_{\Omega }J\left(x^{\prime }\right)\times \frac{x-x^{\prime }}{{\left|x-x^{\prime }\right|}^{3}}dx^{\prime }+ℋ\left(x\right), \end{array}$$

where *ℋ* is magnetic flux density from electrodes, wires, and others. Note that ∇^2^ *ℋ* = 0 in *Ω*. Then MR spectrometer provides the complex *k*-space data *𝒮* that is influenced by *B*_*z*_ in the following way. Given current injection *I*_*j*_, we have

(3) 𝒮j(m,n,z)=∫∫M(x,y,z)eiδ(x,y,z)eiγBz,j(x,y,z)Tc  ×e−i(xmΔkx+ynΔky)dxdy,

where *M* is a conventional MR magnitude image, *δ* any systematic phase artifact, *γ* = 26.75 × 10^7^ rad/T·s the gyromagnetic ratio of hydrogen, and *T*_*c*_ the current pulse width in seconds. By the discrete inverse Fourier transformation of the *k*-space data *𝒮* in (3), we obtain the following complex images:

(4) $$\begin{array}{c} {ℳ}^{+}\left(x,y,z\right)=M\left(x,y,z\right)e^{i\delta (x,y,z)}e^{i\gamma B_{z}(x,y,z)T_{c}}. \end{array}$$

To extract *B*_*z*_ data stably while eliminating systematic phase artifact *δ*, we inject the counter directional current providing the counter part of *ℳ* ^+^ in (4) so that we get

(5) $$\begin{array}{c} {ℳ}^{-}\left(x,y,z\right)=M\left(x,y,z\right)e^{i\delta (x,y,z)}e^{-i\gamma B_{z}(x,y,z)T_{c}}. \end{array}$$

Division of *ℳ* ^+^ by its counter part *ℳ* ^−^ leads to

(6) $$\begin{array}{c} B_{z}\left(x,y,z\right)=\frac{1}{2\gamma T_{c}}\arg\left(\frac{{ℳ}^{+}\left(x,y,z\right)}{{ℳ}^{-}\left(x,y,z\right)}\right). \end{array}$$

MRCDI [12] and early stage MREIT [13–15] use measurements of all three components of **B** = (*B*_*x*_, *B*_*y*_, *B*_*z*_) which require subject rotations inside MR scanner. However, experiences show that these subject rotations are impractical and also cause other problems such as misalignments of pixels. Hence, in order to make the MREIT technique easily applicable to clinical situations, we should use only *B*_*z*_ data for the conductivity reconstruction.

In the harmonic *B*_*z*_ algorithm [5, 16, 17] which is the first constructive *B*_*z*_-based MREIT algorithm, we inject two independent electrical currents *I*_1_ and *I*_2_ through two pairs of surface electrodes *ℰ*_1_^±^ and *ℰ*_2_^±^, respectively, to get two raw *k*-space data. The amount of each injection current is determined by the product of current amplitude and time duration. A modified spin-echo pulse sequence synchronized with the current injection is typically used as described in experimental works [5, 18, 19], and *B*_*z*,1_ and *B*_*z*,2_ are obtained from the formula (6). Then the reconstruction algorithm is based on the following identity:

(7) $$\begin{array}{c} \left[\begin{array}{c} \frac{\partial \ln\sigma }{\partial x}\left(x\right)\\ \frac{\partial \ln\sigma }{\partial y}\left(x\right) \end{array}\right]=\frac{1}{\mu_{0}}{\left(𝔸[\sigma ](x)\right)}^{-1}\left[\begin{array}{c} \nabla^{2}B_{z,1}\left(x\right)\\ \nabla^{2}B_{z,2}\left(x\right) \end{array}\right],\;x\in \Omega , \end{array}$$

where

(8) $$\begin{array}{c} 𝔸\left[\sigma \right]\left(x\right)=\left[\begin{array}{cc} -J_{y,1} & J_{x,1}\\ -J_{y,2} & J_{x,2} \end{array}\right]=\left[\begin{array}{cc} \sigma \partial_{y}u_{1} & -\sigma \partial_{x}u_{1}\\ \sigma \partial_{y}u_{2} & -\sigma \partial_{x}u_{2} \end{array}\right],\;x\in \Omega \end{array}$$

and *J*_*x*,*j*_ and *J*_*y*,*j*_ are the *x* and *y* components of induced current density **J**_*j*_, respectively. We now present the conductivity reconstruction procedure using the harmonic *B*_*z*_ algorithm in CoReHA, which consists of six steps.

**Step 1**

We impress electrical currents *I*_1_ and *I*_2_ through pairs of surface electrodes *ℰ*_1_^±^ and *ℰ*_2_^±^, respectively, and get the *k*-space data *𝒮*_*j*_ for *j* = 1,2 using an MR scanner.

**Step 2**

We produce an MR magnitude image *M* and induced magnetic flux densities *B*_*z*,*j*_ from the *k*-space data *𝒮*_*j*_ for *j* = 1,2 given by (6).

**Step 3**

Using the MR magnitude image *M*, we perform segmentation of ∂*Ω* and *ℰ*_*j*_^±^. Here, we use level-set-based segmentations.

**Step 4**

We set the initial guess *σ* ^0^(**x**, *z*) = 1 in *Ω*.

**Step 5**

We solve the problem (1) with *σ* = *σ* ^0^.

**Step 6**

We solve

(9) $$\begin{array}{c} \nabla_{xy}^{2}\ln\sigma^{1}=\nabla_{xy}\cdot \left(𝔸{\left[\sigma^{0}\right]}^{-1}\left[\begin{array}{c} \nabla^{2}B_{z,1}\\ \nabla^{2}B_{z,2} \end{array}\right]\right)\;\text{in}\;\;\Omega_{z} \end{array}$$

with the boundary condition

(10) $$\begin{array}{c} \nabla_{xy}\ln\sigma^{1}\cdot \nu =\left(𝔸{\left[\sigma^{0}\right]}^{-1}\left[\begin{array}{c} \nabla^{2}B_{z,1}\\ \nabla^{2}B_{z,2} \end{array}\right]\right)\cdot \nu \;\mathrm{on}\;\;\partial \Omega_{z}, \end{array}$$

where ∇_*xy*_^2^ and ∇_*xy*_ are two-dimensional Laplacian and gradient operators on the *xy*-plane, respectively, *Ω*_*z*_ a two-dimensional slice of *Ω*, which is perpendicular to the *z*-axis, and *ν* the two-dimensional outward normal vector to ∂*Ω*_*z*_.

Note that in the harmonic *B*_*z*_ algorithm in [5, 16], Steps 5 and 6 are repeated to get *σ* ^*n*^ from *σ* ^*n*−1^ until it converges. In order to ensure the convergence of the harmonic *B*_*z*_ algorithm, the conductivity values of the subject's boundary should be homogeneous and the contrast of the conductivity distribution is assumed small enough [16, 17]. But, in general, the conductivity distribution of animal and human is quite inhomogeneous [11]. Also, for the completion of the iterative procedure, we have to resolve heavy cost computational issues for 3D forward problem (1), related to, for example, 3D segmentation, 3D mesh generation, and 3D forward problem solver. Fortunately, for the clinical purpose, it is enough to find the scaled conductivity images instead of the true ones because the reconstructed scaled ones reflect the fine details of the true conductivity contrast when the conductivity contrast is low [11]. Hence, in CoReHA, we iterate Steps 5 and 6 only once. Due to the same reason, in Step 5, we solve a simplified problem

(11) $$\begin{array}{c} \nabla_{xy}\cdot \left(\sigma \nabla_{xy}u_{j}\left[\sigma \right]\right)=0\;\text{in}\;\;\Omega_{z}\\ u_{j}=1\;\mathrm{on}\;\;{ℰ}_{j}^{+}\cap \Omega_{z}\\ u_{j}=-1\;\mathrm{on}\;\;{ℰ}_{j}^{-}\cap \Omega_{z}\\ \sigma \frac{\partial u_{j}\left[\sigma \right]}{\partial \nu }=0\;\mathrm{on}\;\;\partial \Omega_{z}\setminus \bar{{ℰ}_{j}^{+}\cup {ℰ}_{j}^{-}}, \end{array}$$

instead of (1). In addition, in Step 6, we adopt the boundary condition

(12) $$\begin{array}{c} \sigma^{1}=1\;\mathrm{on}\;\;\partial \Omega_{z}, \end{array}$$

to replace (10). Note that three-dimensional Laplacian of *B*_*z*_ may not be invalid due to the circumstance of the experiments. Hence, CoReHA supports for both 2D and 3D Laplacian of *B*_*z*_.

## 3. CoReHA 1.0: For Multilateral Studies

CoReHA implements Step 2 through 6 of the harmonic *B*_*z*_ algorithm since Step 1 is about the experiment using an MR scanner and EIT equipment. In this section, we briefly describe each step of CoReHA 1.0 for understanding the software package on the whole.

After an MREIT experiment, one can perform the phase extraction process by applying fast Fourier transform in Section 2 as well as obtain the MR imaging by taking magnitude of *ℳ* ^±^. For this computation, we use FFTW [20], one of the well-known fast Fourier transform libraries, for obtaining (4) from (3). Since *B*_*z*_ in (6) is wrapped due to the branch cut of argument operator, we apply the Goldstein's algorithm [21] for two-dimensional phase unwrapping. Note that even if *B*_*z*_ data is continuous in the *xy*-plane given by phase unwrapping, we do not guarantee the continuity along the *z*-direction. In order to verify the continuity of *B*_*z*_ along the *z*-direction, CoReHA supports a verification tool. Hence, if the verification fails, one should drop (∂^2^/∂*z* ^2^)*B*_*z*_ in ∇^2^ *B*_*z*_ for preventing the artifact coming from wrong Laplacian calculation.

In order to get the imaging domain, we have employed statistically reinstating method (SRM) [22] on MR image, which is a level-set-based segmentation method. After the boundary of the object including electrodes is segmented by SRM, one can operate additional manual work to configure the object without electrodes or to modify the local geometry. After segmentation, one can generate the triangle mesh for the numerical computation. For the triangulation, we adopt the Triangle [23] which is a well-known open source software for the two-dimensional triangulation.

For the computation of (11) and (9), we implement the standard *𝒫*_1_ finite element method and apply the conjugate gradient (CG) method for matrix inversion. The size of generated triangulation is automatically determined by given geometrical information and its size is small enough to cover the pixel size. Of course, one can adjust the size of triangulation for one's own purpose.

For the details of CoReHA 1.0, see [3].

## 4. CoReHA 2.0: For Better Imaging Quality

In this section, we describe in detail new features in the second version of CoReHA for dealing with *in vivo* MREIT experiments.

### 4.1. Preprocessing: Ramp Preserving Denoising

**Figure 2:**
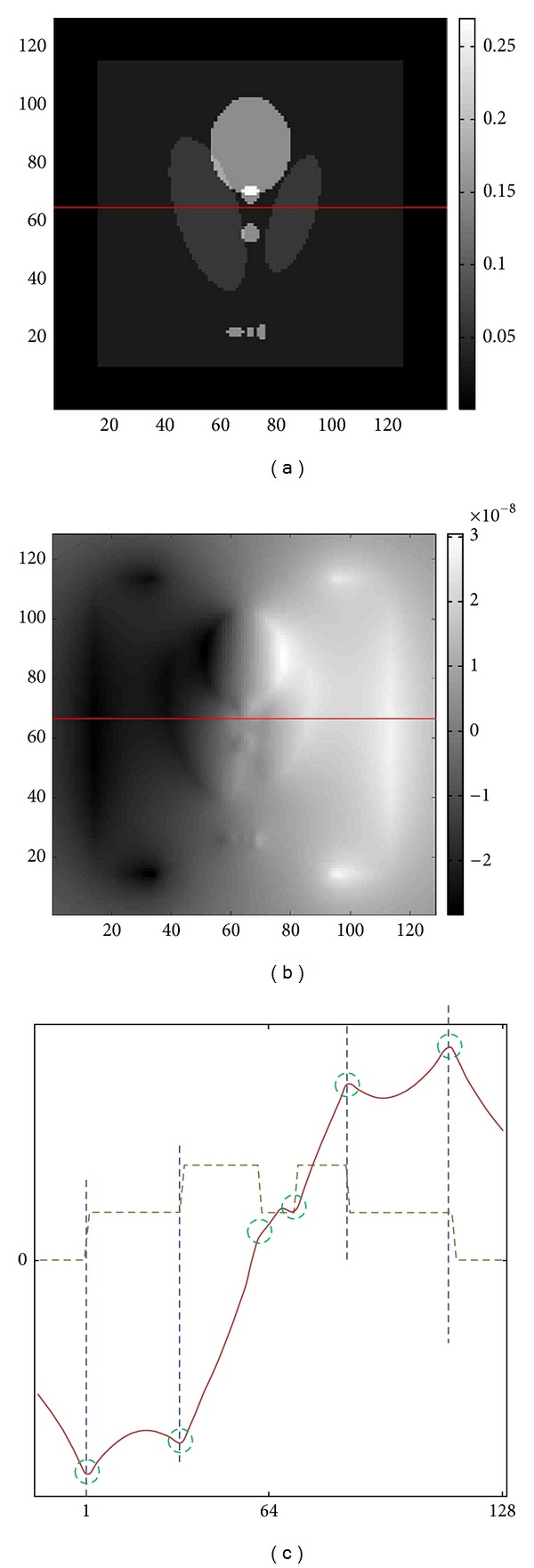
Explanation of ramp structure. (a) Conductivity distribution. (b) Corresponding *B*_*z*_ data generated by the current injection along vertical direction. (c) 1D profiles along the line *y* = 64 which is indicated by the red line in (a) and (b). Green dotted line is the conductivity distribution and the red one is the *B*_*z*_ data. Locations of conductivity change match the ramp structure which is characterised by changes of slopes of the *B*_*z*_ data.

For the safety of the subject in *in vivo* experiments, the amount of injected current *I* should be reduced less than a few milliamperes. The SNR of *B*_*z*_ data is also directly affected by the noise of *ℳ*. Therefore, a proper denoising method is required for obtaining the reconstructed image with reduced noise artifact. Let us consider the reconstruction identity (7) for the derivation of denoising algorithm. Apparently, the change of the conductivity distribution ∇_*xy*_ln⁡*σ* is directly proportional to the Laplacian of *B*_*z*_ data if the matrix *𝔸*[*σ*](**x**) is invertible. When we apply a denoising algorithm, the structure of ramp should be preserved in order to prevent the change of shape or wrong alignment of anomaly location. For our better understanding, we illustrate a simple example using a modified Shepp-Logan phantom. We compute the current density using (11) with the vertically identical conductivity distribution given in Figure 2(a). Applying the Biot-Savart law (2), we obtain the *B*_*z*_ data as shown in Figure 2(b). We can see that the ramp structure of *B*_*z*_ reflects the change of the conductivity distribution as shown in Figure 2(c). In order to keep the ramp structure during the denoising, we adopt a nonlinear diffusion equation method based on structure tensor [9, 24].

For the sake of completeness, we summarise the results in [9, 24]. Let us consider the structure tensor *U* defined by

(13) $$\begin{array}{c} U:=\sum_{i=1}^{2}\nabla_{xy}w_{i}\nabla_{xy}w_{i}^{T}, \end{array}$$

where

(14) $$\begin{array}{c} \left(\begin{array}{c} w_{1}\\ w_{2} \end{array}\right):=\nabla_{xy}B_{z}=\left(\begin{array}{c} \partial_{x}B_{z}\\ \partial_{y}B_{z} \end{array}\right). \end{array}$$

To reduce the noise, we solve the following nonlinear PDE cooperating with the structure tensor *U*:

(15) ∂tBz(x,t)=∇xy·(g(U(x,T2))∇xyBz(x,t))          in  R×(0,T1],(g(U(x,T2))∇xyBz(x,t))·n=0 on⁡  ∂R×(0,T1],Bz(x,0)=Bz(x) on⁡  R,

with

(16) $$\begin{array}{c} \partial_{\tau }u_{ij}\left(x,\tau \right)=\nabla_{xy}\cdot \left(g\left(U_{s}\right)\nabla_{xy}u_{ij}\left(x,\tau \right)\right)\;\text{in}\;\;R\times \left(0,T_{2}\right],\\ \left(g\left(U_{s}\right)\nabla_{xy}u_{ij}\left(x,\tau \right)\right)\cdot n=0\;\mathrm{on}\;\;\partial R\times \left(0,T_{2}\right],\\ u_{ij}\left(x,0\right)={\left(\sum_{k=1}^{2}\nabla_{xy}w_{k}(x,t)\nabla_{xy}w_{k}{\left(x,t\right)}^{T}\right)}_{ij}\;\mathrm{on}\;\;R,\\ g\left(U\right)=\frac{1}{\sqrt{1+\Lambda }}v_{\Lambda }v_{\Lambda }^{T}+\frac{1}{\sqrt{1+\lambda }}v_{\lambda }v_{\lambda }^{T}, \end{array}$$

where *R* is a two-dimensional region for the slice of *B*_*z*_ data, *u*_*ij*_ is the (*i*, *j*) element of *U*, Λ and *λ* are maximum and minimum eigenvalues of *U*, respectively, *v*_Λ_ and *v*_*λ*_ are corresponding normalized eigenvectors, and *U*_*s*_ ≡ *G*_*s*_∗*U* is the (element by element) convolution of *U* with the two-dimensional Gaussian kernel *G*_*s*_ with a standard deviation *s*. In the new version of CoReHA, the user only considers the parameter *T*_1_, the total diffusion time of the denoising algorithm. Through extensive numerical studies [9, 24], we figured out that the denoising results are robust to the tensor regularization time *T*_2_. The noise level of *B*_*z*_ affects only *T*_1_. Consequently, we fix *T*_2_ = 2 in this version, which seems to be enough time to regularize the diffusion tensor. Note that the denoising algorithm is only valid when the user considers two-dimensional Laplacian of *B*_*z*_ since the diffusion process is on the *xy*-plane. For more details, see [9].

### 4.2. Preprocessing: Harmonic Inpainting Using MR Data

**Figure 3:**
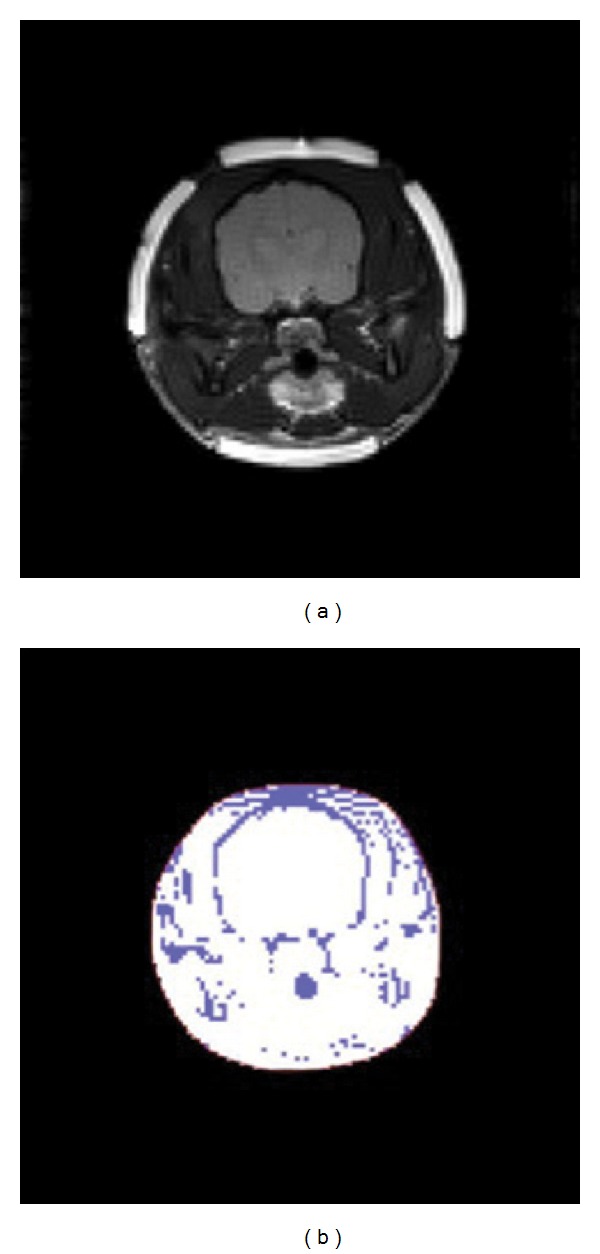
Defected region select using threshold (a) MR image and (b) defected region by the thresholding (purple color).

Let us revisit (6). In a local region of *Ω*, where |*ℳ* ^±^| ≈ 0, *B*_*z*_ is defected severely by the amplification of noise due to the division procedure. In the previous version of CoReHA to overcome this trouble, a manual segmentation tool was provided for the extraction of the problematic region with the harmonic inpainting algorithm [10] to solve the Poisson equation. However, the manual segmentation causes user-dependent reconstruction results. For dealing with this situation, we assume that the defected region has low MR magnitude and its conductivity distribution is homogenous so that ∇^2^ *B*_*z*_ = 0. Then, we automatically select the defected region by taking a threshold for the MR data.  Figure 3 shows an example for the selection of the defected region by thresholding. In the new version of CoReHA, we consider the 10% of maximum magnitude of MR data as a threshold. Incorporating the ramp-preserving denoising, we additionally apply isotropic diffusion on the defected region within enough duration. The user does not need to consider how well the defected region is segmented; therefore, it guarantees the robustness of the conductivity reconstruction without user dependency.

### 4.3. Reconstruction: Local Harmonic *B*_*z*_ Algorithm

**Figure 4:**
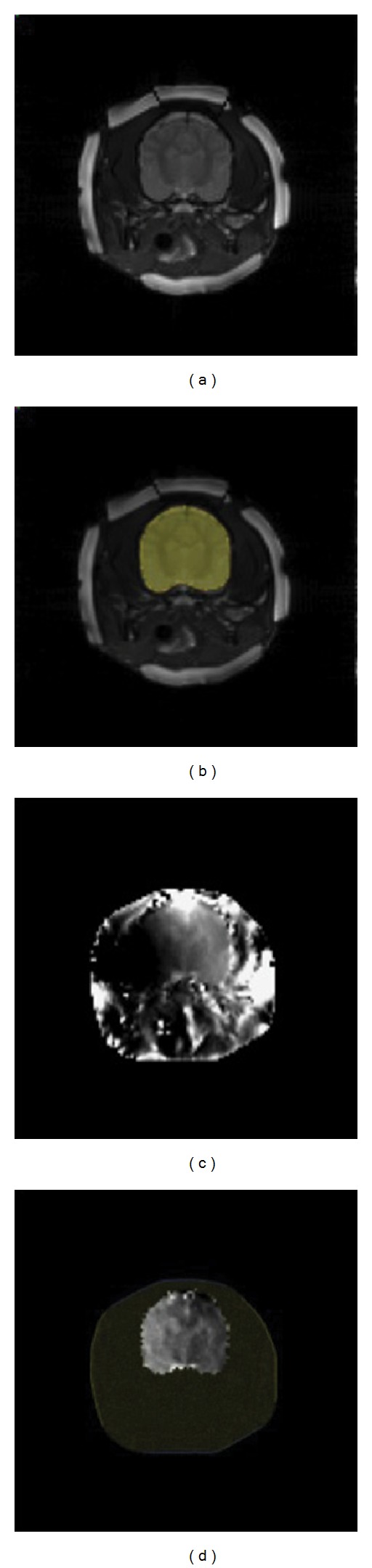
Local harmonic *B*_*z*_ algorithm: (a) MR image, (b) selected interested region, (c) conductivity reconstruction on the whole domain, and (d) conductivity reconstruction using the local harmonic *B*_*z*_ algorithm.

We support a modified local harmonic *B*_*z*_ algorithm [11] for the successful reconstruction by excluding the defected region directly. Let *D* be a local region without defection which attracts our interests. Then, we locally reconstruct the conductivity by solving (9) and (10) on the local region *D* instead of the whole domain *Ω*. In order to solve (9) with (10), we use a *𝒫*_1_ finite element method on a selected triangle mesh and apply conjugate gradient (CG) method for matrix inversion. CoReHA 2.0 supports a user interface to draw and manipulate a polygonal local region by adding or removing points. It is useful when the user wants to see a small local change of conductivity on the local region such as brain or pelvis. In Figure 4(c), we see that the reconstructed conductivity is severely contaminated by locally magnified noise. But, Figure 4(d) shows the successful reconstruction of local brain region by the local harmonic *B*_*z*_ algorithm. 

### 4.4. Additional Tools

Due to the different physical properties between water and fat, the measured MR signal can be shifted in the fat region, since the MR scanner assumes that spin isochromats precess at the Larmor frequency of water. Therefore, the MR signal may become void, and overlapped images are produced in the animal/human experiments. To overcome this difficulty, new version of CoReHA contains the chemical shift correction using 3-points Dixon method [25]. In the *k*-space data conversion, one can manipulate the MR signal using the user interface which provides separated water and fat images of MR. For more details, see [25].

**Figure 5:**
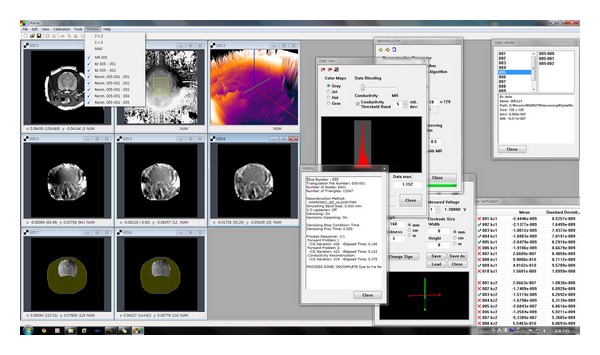
Snapshot of CoReHA 2.0.

Furthermore, we implemented many useful features to help our understanding of MR, *B*_*z*_, and conductivity images, for example, magnification windows and advanced alignment and resizing of windows.  Figure 5 is a snapshot of CoReHA 2.0, that shows newly introduced features.

## 5. Conclusions and Future Works

In this paper, we presented new features in a software package, called CoReHA 2.0, to deal with the data from the *in vivo* stage of MREIT. In this version, we enhanced the preprocessing procedure based on the ramp preserving denoising and harmonic inpainting with MR data to increase SNR of *B*_*z*_ data in the *in vivo* experiment. Also, we provided a user interface for the local harmonic *B*_*z*_ algorithm for the reconstruction on the locally interested region. The performance results of suggested algorithms and methods are given in [26]. Note that CoReHA reconstructs the contrast image of conductivity. For the medical diagnosis, the contrast image is enough. However, recently the necessity for the true conductivity came to the fore. For the true conductivity image reconstruction, we need a subject-dependent 3D segmentation and modeling tools, 3D denoising algorithm, and 3D finite element solver and higher order methods for more accurate computation.
